# Dynamic systems approaches and levels of analysis in the nervous system

**DOI:** 10.3389/fphys.2013.00015

**Published:** 2013-02-05

**Authors:** David Parker, Vipin Srivastava

**Affiliations:** ^1^Department of Physiology, Development and Neuroscience, University of CambridgeCambridge, UK; ^2^Centre for Neural and Cognitive Sciences, School of Physics, University of HyderabadHyderabad, India

**Keywords:** neuronal network, criticality, spinal cord

## Abstract

Various analyses are applied to physiological signals. While epistemological diversity is necessary to address effects at different levels, there is often a sense of competition between analyses rather than integration. This is evidenced by the differences in the criteria needed to claim understanding in different approaches. In the nervous system, neuronal analyses that attempt to explain network outputs in cellular and synaptic terms are rightly criticized as being insufficient to explain global effects, emergent or otherwise, while higher-level statistical and mathematical analyses can provide quantitative descriptions of outputs but can only hypothesize on their underlying mechanisms. The major gap in neuroscience is arguably our inability to translate what should be seen as complementary effects between levels. We thus ultimately need approaches that allow us to bridge between different spatial and temporal levels. Analytical approaches derived from critical phenomena in the physical sciences are increasingly being applied to physiological systems, including the nervous system, and claim to provide novel insight into physiological mechanisms and opportunities for their control. Analyses of criticality have suggested several important insights that should be considered in cellular analyses. However, there is a mismatch between lower-level neurophysiological approaches and statistical phenomenological analyses that assume that lower-level effects can be abstracted away, which means that these effects are unknown or inaccessible to experimentalists. As a result experimental designs often generate data that is insufficient for analyses of criticality. This review considers the relevance of insights from analyses of criticality to neuronal network analyses, and highlights that to move the analyses forward and close the gap between the theoretical and neurobiological levels, it is necessary to consider that effects at each level are complementary rather than in competition.

“Any one will renovate his science who will steadily look after the irregular phenomena. And when the science is renewed, its new formulas often have more of the voice of the exceptions in them than of what were supposed to be the rules.”

William James (1897)

## Introduction

Life is said to occur at the border between order and chaos (Macklem, 2008): it requires stability, traditionally expressed in terms of homeostatic principles, with flexibility and adaptability at the micro (molecular and cellular) and macro levels (networks, organisms). Schrodinger (1944) defined life as the passage of encoded material from parent to offspring, and the spontaneous emergence of self-organized order. The former aspect is being dealt with quite successfully by biochemistry and molecular biology; the latter aspect is for physiology to address and it remains open. Significant mechanistic insight has been obtained by reducing systems to their components (i.e., assuming proportionality and superposition, that systems are sums of their parts). While this approach has driven some major advances in genetics, developmental biology, and cellular physiology (obvious examples in the nervous system are the Hodgkin–Huxley analysis of the action potential and Katz's quantal model of synaptic transmission), it is unlikely to be successful at the systems physiology level where outputs depend on non-linear interactions between components parts. Despite advances, the reductionist/holism split continues, and while the examples given above argue against the claim that reductionism has not revealed much of the nature of biological complexity (Pattee, 1979), it is the case that these analyses do not explain higher-level functions.

Among physiological systems the brain in particular defies explanation, at least to a point where we can explain normal and abnormal behavior in mechanistic terms related to underlying cellular processes. Understanding the dynamics of nervous system activity requires insight into effects occurring at multiple spatial and temporal scales that differ by orders of magnitude. In practical terms there are three spatial divisions: microscopic (molecular/cellular/synaptic), mesoscopic (network and associated measures of local interactions), and macroscopic (whole system or behavior). The criteria that need to be met to satisfy definitions of understanding can differ depending on the level of analysis. Microscopic analyses (molecular biology, single cell imaging, electrophysiology) provide insight into the properties of single molecules, cells, or synapses but often give little insight into their higher functional relevance; whereas mesoscopic or macroscopic analyses [monitoring network activity (e.g., imaging), EEG, PET, fMRI] provide insight into activity in different brain areas relevant to different tasks but give little or no insight into their underlying mechanisms. Despite their individual weaknesses analyses tend to focus on a specific level. The question of how higher-level outputs (network activity, behaviors) are generated from lower-level properties (cells and synapses) has been considered for decades and still exists. The major problem is linking across levels in the nervous system, for example, in the way that NAD and other enzymes are linked as functional components of the Kreb's cycle, which is in turn linked as a component of metabolism. Where strong claims to these mechanistic links have been made in the nervous system they are either demonstrably in error or underdetermined by the data and reflect the belief that the level of explanation given is sufficient (see Dudai, 2004; Parker, 2006, 2010).

## The problem of linking between micro and macro levels in biological networks

Take as an example a neuronal network, that is a population of functionally heterogeneous neurons, connected to each other through functionally heterogeneous cell-specific (i.e., not all-to-all) synaptic connections, with the additional potential for interactions resulting from electrical or chemical ephapses or fields (Jefferys, 1995; Weiss and Faber, 2010). This reflects biological but often not artificial neural networks. Similar considerations apply to interactions between regions of the nervous system (e.g., Bullmore and Sporns, 2009), where each region is actually a neuronal network but can be treated as a component. Assume, following traditional neurobiological criteria, that understanding implies the ability to explain the spatial and temporal aspects of the networks activity in terms of the activity of its cellular and synaptic components (see Selverston, 1980; Yuste, 2008; Parker, 2010). The fundamental first step according to these criteria is to identify the network neurons: while this seems trivial, even this initial step can be difficult as network neuron identification criteria can introduce errors of inclusion or exclusion (see Parker, 2010), and non-neuronal elements (glia) may also contribute to network function (Araque and Navarrete, 2010). Secondly, the network connectivity must be determined. This requires the identification of monosynaptic (direct) connections between identified neurons, an approach that ideally needs the simultaneous recording and manipulation of activity in the presynaptic and postsynaptic neurons. Caution is needed here as synaptic function is complicated by various microlevel synaptic arrangements (e.g., dendro-dendritic interactions, graded or electrical synapses, ephapses, glial signaling), and the history of network connectivity analyses highlights errors that can arise from approaches still used routinely (e.g., temporal correlation) that erroneously claim to unequivocally identify monosynaptic connections (see Berry and Pentreath, 1976; Parker, 2010). Much is rightly made of the need to describe network interactions (e.g., Yeh et al., 2010), although there can be a mismatch between what is meant by connectivity from lower-level synaptic analyses and higher-level statistical approaches. Identifying the direct or indirect interactions between components is essential to network understanding as functional inferences are based on the assumed organization. Thirdly, from this description of the network architecture the functional properties of cellular and synaptic components must be identified. This is a daunting task: there are in excess of 200 transmitter substances (Thomas, 2006) whose temporal and spatial effects at the synapse are varied, activity-dependent, non-linear, and cell specific. The voltage-dependent ion channels that determine the non-linear resting and active properties of neurons form a superfamily of at least 143 genes (Yu et al., 2006), with further diversity and functional variability of cells resulting from alternative splicing, posttranslational modifications, and varying combinations of channel subunits (Gutman et al., 2005; see Parker, 2010). This variability means that even neurons and synapses considered as belonging to single populations or classes on the basis of particular molecular or anatomical markers cannot be reduced to a collection of functionally homogeneous units. This makes the functional description of even small networks far more difficult than a description of the network architecture.

**Figure 1:**
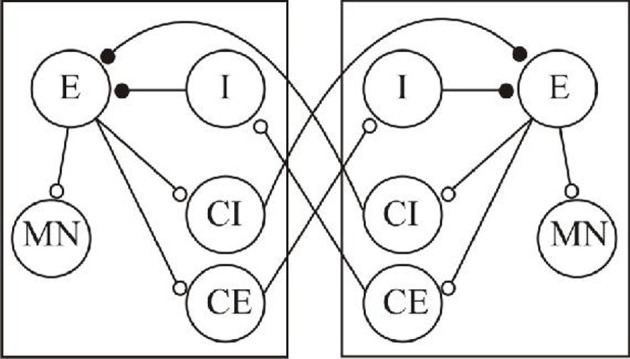
**A simplified scheme of a basic organization of spinal cord locomotor networks.** The two boxes represent antagonistic networks (“half-centers” that control, for example, activity on the left and right sides of the body). The circles represent neuronal populations, and the lines synaptic connections. The small open circles indicate excitatory (glutamatergic) synaptic connections, the filled circles inhibitory (glycinergic) connections. E, excitatory interneuron; MN, motor neuron; I, inhibitory interneuron; CI, crossing inhibitory interneuron; CE, crossing excitatory interneuron.

This brief outline highlights several problems associated with the analysis of neuronal networks. Firstly, it is currently impossible to perform a detailed microlevel characterization in systems of more than a few tens of components: analyses become impractical as the number of components and interactions increase (the “tyranny of numbers” and the “curse of dimensionality,” respectively: see Koch (2012) for a recent concise review of analytical complexities). Analyses thus have to change as systems increase in size to focus on cellular (spiking) or system outputs and correlations between active components rather than the details of these components or interactions (e.g., Schneidman et al., 2006). This is akin to statistical mechanics, and ignores component properties to examine global effects (i.e., parameter functions rather than parameter values). However, global outputs reflect extensive microscale analogue sub- and suprathreshold cellular and synaptic processing, and these effects do matter because the neuron is not “simple” or “repetitive” (cf. West, 2010), lower-level properties are not hidden, irrelevant, or homogeneous (i.e., defined populations cannot be reduced to single functional units), and activity in single cells can have significant functional effects, not only in smaller networks where they are endemic (e.g., Selverston, 2010), but also in systems where only population-level responses were assumed (Houweling and Brecht, 2008). Even though macroscopic or mesoscopic analyses can describe network outputs, as similar outputs can result from markedly different lower-level effects (Prinz et al., 2004; see Figure 1) these analyses do not allow confident assumptions to be made of the network organization or mechanisms, features that are essential for any rational manipulation of real systems.

While lower-level analyses highlight the need for microlevel information, even if it was universally accepted (which it is not) that microlevel information was necessary for understanding, it is not sufficient. A complete library of individual components would not constitute understanding of a system that included non-linear positive and negative feedback loops between component parts and circular interactions between network activity and activity-dependent cellular and synaptic properties. These cannot be understood by analyzing components individually or in the absence of ongoing network activity, even if emergent or ephaptic or field-like effects are ignored (Jefferys, 1995; Weiss and Faber, 2010). If the latter effects are present even targeted perturbations of network components with much greater specificity than is currently possible would not allow simple interpretations of functional effects, as any perturbation will alter field effects and change the properties of supposedly unaffected components.

One potential approach is to identify network motifs (Simon, 1962; Sporns et al., 2005; Koch, 2012) or “building-blocks” (Getting, 1989), which chunk elements of the network into defined functional units and thus reduces the number of components to be analyzed (Koch, 2012). However, the identification of a structural motif does not determine its function (e.g., Ingram et al., 2006; Qian et al., 2011), as this also depends on the properties of the components and their connections (Selverston, 1980; Getting, 1989; Parker, 2003; Song et al., 2005; Perin et al., 2011). Sporns and Kotter (2004) using a statistical analysis of neuroanatomical data highlight that there is a small number of structural motifs, and suggest that there will be great diversity of functional motifs. This is something that is known from lower-level analyses (e.g., Arshavsky et al., 1993; Elson et al., 2002; Prinz et al., 2004), and shows that structural motifs alone cannot infer a set function. Motif interactions may, however, follow rules that allow inferences of connectivity and connection properties (e.g., Perin et al., 2011). As neuronal and synaptic properties vary widely within single classes and within and between networks these properties will still need to be examined in specific cases. Nevertheless, the identification of conserved motifs could at the very least direct experimental analyses in systems that would otherwise be intractable (see Koch, 2012). Several motifs are already known: feedforward and feedback excitation can amplify effects and increase excitation and synchronous firing among functionally related cells; feedback, reciprocal, or lateral inhibition can underlie mutual inhibition responsible for selection or patterning of activity; and cyclical inhibition can produce oscillatory activity, the number of phases depending on the number of cells in the ring (Szekely, 1965). These structural motifs may have been a necessary stage in the evolution of the nervous and other complex systems (Simon, 1962), and as such may serve as useful guides for analysis. However, all are subject to changes depending on the functional properties of the components. Striking examples are that reciprocal excitation can lead to inhibition rather than excitation (Egelhaaf and Benjamin, 1982), and reciprocal inhibition can lead to synchronous rather than alternating activity (Elson et al., 2002). We must be careful when using terms like building-block or motif that we avoid the inherent danger of reifying components as fixed assumed units that sum to give a particular output.

These aspects emphasize the need for novel experimental and analytical approaches in network analyses. Latest techniques are often lauded as overcoming problems, but available experimental approaches still have to go far beyond the claimed capabilities of even the most sophisticated techniques to understand even modest-sized simpler networks (Parker, 2010). Useful insights can come from the cross-fertilization of ideas between different fields. Analytical approaches have often been inspired by the physical sciences. Below we discuss dynamical systems approaches and their application to varied physiological systems, and highlight the potential relevance of these insights to analyses of neuronal networks.

## The dynamical systems approach to networks

It has been argued that mechanistic (cellular and synaptic) explanations in the nervous system can be replaced at higher levels by dynamical systems theory (see Haken, 2006; Werner, 2007). This substitutes analysis of component properties for predictions based on appeals to the sufficiency of universality classes: if a model describes a phenomenon and predicts future effects then it is considered to explain the actual system (see Kaplan and Craver, 2011 for a critique of this view).

The dynamical systems approach in neuroscience has been highlighted many times [for example, Turing (1950), Ashby (1962), Friston (2000), Haken (2002), Freeman (2005), Tognoli and Kelso (2009)]. Complexity has emerged as one of the main analytical approaches (Simon, 1962; Pattee, 1979). Although there has not, and may never be a generally accepted definition of complexity (see Pattee, 1977), in the nervous system it has been defined as the structure in a systems dynamics: complexity is high in systems where different parts can act separately while still being interdependent (Tononi et al., 1994). As complexity analyses aim to examine how interactions between component parts generate collective global behaviors, it could offer a bridge between micro and macro level effects in suggesting how system parameters influence system function. An advantage of looking at a problem as a critical phenomenon is that insight can be obtained with the help of one or a few order parameters. This offers an alternative to network analyses using computer simulations, where although larger, physiologically relevant, numbers of components are being modeled in increasing detail (although in many cases parameters are still often poorly defined, and reduced to mean, assumed, or extrapolated values), and the simulations can recreate features of the actual system, but as they become more detailed they can prevent understanding of the underlying mechanisms (see Greenberg and Manor, 2005).

## Complexity in neural systems

Physiological outputs are typically studied in relation to their instantaneous frequency and amplitude. However, various scaling techniques inspired by analyses in physical systems have revealed long-range power-law correlations in apparently random fluctuations that suggest that processes at particular temporal or spatial scales are linked to those at other scales (scale-invariance; Goldberger et al., 2002; West, 2010). Scale invariance is assumed to be a natural property of self-organized systems and suggests a memory-like process where outputs do not simply depend on the recent state of the system but also on the state at earlier times (see Huang, 1987). In equilibrium systems, scale-invariance appears at the critical point of a second-order phase transition (Chialvo et al., 2008). However, natural systems are out-of-equilibrium and the common appearance of scale-invariance in such systems is still not well-understood (Bailly and Longo, 2008; Yeh et al., 2010).

Self-organized criticality (SOC) is a fractal-related feature of dynamical systems where the macroscopic behavior of a system arises from the interactions of its component parts. This results in non-equilibrium phase transitions that depend on the intrinsic dynamics of the system rather than requiring external tuning (Bak et al., 1987). In its simplest form, a SOC system is a driven dissipative system consisting of a medium with disturbances propagating through it that cause the medium to be modified in such a way that it develops into a critical state (Flyvbjerg, 1995). SOC is considered a key concept underlying complexity in natural systems, and was introduced to describe how dynamical systems intrinsically organize at critical states from which energy fluctuations of all sizes occur.

SOC has been associated with various patterns of activity in real and artificial neural systems (Usher et al., 1995; Linkenkaer-Hansen et al., 2001; Beggs and Plenz, 2003; Eguiluz et al., 2005; El Boustani et al., 2005; Freeman, 2005; see Werner, 2007, 2010 for a detailed list of effects and the variability between studies). This suggests a state-dependent influence that extends far beyond that usually considered in neuronal analyses. State-dependent effects have generally received little attention (see Buonomano and Maass, 2009), but the presence of long-range correlations would make them far more difficult to identify and track, and a consideration of these effects could require changes in the design of conventional experimental approaches. While analyses of SOC have generated some interesting insights (see below), in relating SOC to neuronal networks we are faced with a mismatch in the language used to describe effects at the micro and macro levels: the latter are statistical and mathematical (scaling exponents, wave patterns, attractors, bifurcations, phase transitions, Lyapunov exponents), the former biological (neuron, synapse, spikes, transmitters). To link between these we will need to know what to look for in neurobiology as an example of a Lyapunov function or the scaling exponents of power-law relationships. The latter are assumed to be related to underlying physiological mechanisms and network architectures, but these mechanisms are not specified by the analyses. Long term correlations in various aspects of cellular or network activity could reflect feedback loops that adjust outputs as a function of previous activity, but again the underlying mechanisms and functional relevance of the correlations is unknown.

Despite these issues SOC could offer several advantages to the analysis and function of perceptual, cognitive, and motor networks. It could prevent entrainment that locks the system into sub-critical activity (which limits potential responsiveness) or supercritical activity (which limits control; see Kelso, 1991; Fingelkurts and Fingelkurts, 2006), and long-range correlations could facilitate the rapid state transitions needed for the processing of neural signals that may span several orders of magnitude. SOC may also help nervous systems to overcome the “stability-plasticity dilemma” (Abraham and Robbins, 2005), the apparently conflicting requirement of stability (order) with flexibility and plasticity. This requires that changes occur while the system is kept within certain limits, a property that exists with higher probability in systems operating at or close to criticality (see Aldana et al., 2007). Finally, SOC may allow interacting components to organize into functional systems according to local system rules rather than requiring the prior specification or external tuning of each component. This could make systems fault-tolerant as aberrant changes in component values during development or as a result of injury or random cell loss could be compensated for by changes in other components (for examples from neurobiology see Prinz et al., 2004; Marder and Goaillard, 2006; see West, 1990 for a formal analysis of how scale-free effects prevent the propagation of the errors that would be associated with classical scaling effects).

In causing parameters to be near critical values the system is always near a phase transition. To understand network organization and function the critical points and associated order parameters (e.g., the correlation length) that cellular and synaptic components work toward need to be identified rather than the components themselves, thus potentially reducing the computational demands on neuronal network analyses (Koch, 2012). However, in terms of linking to lower-level mechanisms the focus on critical points simply pushes the question back: again, what is the physiological basis of a critical point or attractor? These properties need to be reflected in the features of neuronal networks: that is, identified classes of neuronal and non-neuronal elements connected to each other through specific monosynaptic and polysynaptic pathways; that act with defined cell-specific cellular and synaptic properties; potential field-like effects arising from global or local activity within the network; and the potential for activity or transmitter-mediated modulation of these functional properties. Effort spent relating analytical terms to potential neuronal mechanisms and organizations would be a significant step toward closing the gap between these levels of analysis.

Support for the idea that nervous systems and neuronal networks are critical systems comes from the seemingly ubiquitous power-law scaling from the molecular to behavioral levels (see Werner, 2010). However, this conclusion remains controversial and data obtained using similar experimental and analytical approaches can produce contradictory results (e.g., Klaus et al., 2011; Dehghani et al., 2012). Scaling exponents that are assumed to reflect underlying physiological mechanisms and network architectures (but do not specify these features) can also differ in analyses of supposedly single phenomena. For example, in a study of epilepsy patients Worrell et al. (2002) showed that scaling exponents differed for each individual, thus predicting different underlying mechanisms. As targeting treatments will require insight into these mechanisms or their causes, the description alone could be argued to provide little insight. Conversely, similar scaling exponents can be generated for diverse phenomena with different underlying mechanisms (Ivanov et al., 2009; Stanley, 1999). Despite these issues, the power-law is claimed as one of the few universal mathematical laws of life, and references have been made to a new “law of nature” and “universal architecture” that link complex systems across the natural, social, and engineering sciences (Wolf et al., 2002). While the endemic nature of power law distributions could suggest a fundamental property of multicomponent systems, the ease with which they can be fitted to experimental data has led to doubts over their function and origin: thus, power-law relationships have been suggested to be artefacts due to thresholding effects applied to stochastic processes or a combination of exponential processes (Reed and Hughes, 2002; Bonachela et al., 2010; Touboul and Destexhe, 2010; Stumpf and Porter, 2012).

Stumpf and Porter (2012) highlight the need for caution and empirical support for analytical results applied to physiological systems, as the theories underlying power-law behavior derive from infinite systems, but the systems and data examined are finite. They state, “as a rule a candidate power law should exhibit an approximately linear relationship on a log-log plot over at least two orders of magnitude on the *x* and *y* axes,” a requirement they say that rules out most data sets, and almost all data from biological analyses. Varotsos et al. (2011) have recently shown that dynamic features of complex systems can be derived from their time series in terms of a “natural” time (χ_k_ = k/N), an index for occurrence of the kth event in a time series comprising *N* events. They derived κ_1_ from the Taylor expansion of the power spectrum, as the variance of the natural time and demonstrated that when κ_1_ converges to 0.07 a variety of dynamical systems approach criticality where, for instance, a typical time dependent correlation length ξ would behave as ξ∝χ^1/z^ where z is the dynamic critical exponent. While this has been applied to critical phenomena from geophysical systems and 2D Ising models that exhibit SOC, it will be necessary and instructive to investigate these effects in biological systems where constraints on data series are more significant. Techniques to analyze phase transitions in statistical mechanics also generally have reversibility at the microscopic level and irreversibility at the macroscopic level. Statistical mechanics seeks to establish connections between these two scenarios, and also describes the approach to equilibrium. The question is how do we ensure that these two requirements are fulfilled? Statistical mechanical approaches generally appeal to the ergodicity principle but the latter ceases to hold in systems in a critical state. This does not mean that statistical mechanics fails when systems are in a critical state, or the above mentioned two requirements stop being necessary. If statistical mechanics is done by setting up “master (or rate) equations” then the two requirements are fulfilled, and critical systems can be handled (see Kadanoff and Swift, 1968). Statistical mechanics typically deals with systems containing large numbers of a single or a few types of randomly interacting components, whereas biological systems contain relatively few copies each of multiple components with specific interactions (Hartwell et al., 1999), and assumptions of homogeneity in component properties are not supported (see Soltesz, 2006). It may thus be worthwhile attempting master equation approaches to biological systems.

Non-equilibrium approaches may also be better applied to biological systems, even though fundamental uncertainties remain over their application (Yeh et al., 2010). A core problem is that it is not clear how the organization and operation of neuronal networks relate to the terms used in these analyses. Even when a fit is robust the lack of direct insight into the actual network architecture and function remains problematic (see Fox-Keller, 2005). Work on criticality does show that certain system properties can be understood without involving the details of the system, but not what properties. Watts (2003), who has extensively used statistical analyses of various networks, has said, “For any complex system, there are many simple models we can invent to understand its behavior. The trick is to pick the right one. And that requires us to think carefully—to know something—about the essence of the real thing” (cited in Fox-Keller, 2005). This highlights a familiar circularity to experimental analyses of neuronal networks: the interpretation of effects obtained using experimental tools (e.g., gene knockouts) requires prior knowledge (or more often assumptions of knowledge) of system components, of what the techniques do, and of what the data shows: the results obtained using these assumptions are then used as evidence to support the initial assumption (see Parker, 2010).

## Complexity and disease

The control of physiological systems to ensure their proper functioning is a principal goal of medicine. Complexity is claimed to be a better indicator of physiological functions in health and disease (West, 2010), but does it offer new insight or a new description? That it is the former is clearly suggested by the findings in several systems that disease states reflect a loss of complexity, not the loss of regularity. This is encapsulated in the idea of “dynamic disease” (Mackey and Glass, 1977), and could reflect the reduced interaction between network elements (Pincus, 1994). The breakdown of complexity is generally associated with reduced variability, a constant dominant frequency (mode-locking), and highly regular periodic activity [this has been shown in series of heartbeat intervals and breathing (e.g., Cheyne-Stokes); see Goldberger et al., 2002], but can also be associated with increased variability and with a loss of long-term correlations (i.e., random rather than correlated fluctuations; Hausdorff et al., 1998). The nervous system is arguably unique in having so many functional disorders that despite decades of investigation lack discrete anatomical or physiological markers (generally truer for psychiatric rather than neurological disorders). This could support a focus on disease states where changes in control parameters rather than in component properties lead to abnormal dynamics (Stam and van Straaten, 2012). The appearance of long-range correlations on different time scales in various physiological systems has encouraged analyses that have shown similar long-range effects in neurological and psychiatric disease (Linkenkaer-Hansen et al., 2005). The presence of these correlations is potentially problematic for neuroscience as commonly used analytical approaches are not sufficient for modeling discontinuous, non-homogeneous and irregular processes in self-organizing systems (see Freeman, 2005).

Consider Parkinson's disease, a prevalent neurological disorders that exhibit great variability between and within individuals. It is associated with a classical triad of symptoms that include tremor, bradykinesia, and akinesia (slowness or loss of motor function). The cardinal pathological feature is degeneration of dopaminergic neurons in the substantia nigra pars compacta (Hornykiewicz, 2006). However, there are no symptoms of Parkinson's disease until approximately 80% of the dopaminergic neurons are lost (Bezard et al., 2003). The non-linear relationship between dopamine levels and symptoms could suggest either degeneracy or redundancy in the dopaminergic system (this seems unlikely given the energetic demands of developing and maintaining cell populations), or that the system is able to compensate for differences in dopaminergic inputs to maintain function up to a critical level (i.e., fault tolerance) before the compensation breaks down. Parkinson's disease is suggested to reflect the simplification of a complex dynamic process from a critical to an ordered state (Hausdorff, 2009). In this scheme akinesia (inability to perform movements) reflects a switch to a fixed point of a basin of attraction that restrains behavioral flexibility, and tremor reflects a change from the small aperiodic normal resting tremor to pathological regular large amplitude tremor due to changes in a dynamical regime produced by multiple feedforward and feedback loops between basal ganglia and possibly other networks (Bezard et al., 2003; Hausdorff, 2009).

If disease reflects a loss of network complexity, then it would require an understanding of network-level dynamical disease processes rather than the focus on changes in single components (e.g., dopamine levels), and treatments would need to manipulate complexity arising from dynamic interactions rather than single components (again, dopamine levels in Parkinson's disease). Deep brain stimulation and transcranial magnetic stimulation are the latest treatments being applied to neurological and psychiatric conditions (see Goodman and Insel, 2009). However, there is significant heterogeneity in their efficacy and a narrow window between beneficial and adverse effects of stimulation. In a metaanalysis of deep brain stimulation in Parkinson's disease there was a mean improvement rate of 52% (this primarily reflected improvements in physical function not quality of life; Kleiner-Fisman et al., 2006). The variable success rate may reflect intrinsic differences in patient or disease properties (severity, duration, drug treatment history), but it may also reflect a requirement for better understanding and application of dynamic inputs rather than current stimulation regimes that use fixed values of amplitude, width and frequency adjusted for each patient (Volkmann et al., 2006). Dynamical approaches have been introduced in intensive care: an inverse power-law spectrum rather than a fixed regular artificial respiration rate was used to drive a variable ventilator rhythm with a resulting increase in arterial oxygenation (Mutch et al., 2000), supporting the utility of this approach.

## Complexity, variability, and homeostasis

Healthy physiological systems are assumed to homeostatically reduce variability and settle into constant equilibrium-like states, but in many cases even under steady-state conditions physiological outputs are not constant but fluctuate about some mean value (Buchman, 2002; Goldberger et al., 2002). Non-stationary and non-equilibrium systems are not suitable for the analytical approaches typically applied to the nervous system, which assume linearity, stationarity, and equilibrium-like conditions and use probabilistic averaging methods to exact solutions (e.g., mean, variance, power spectra analyses). Experimental procedures that incorporate averaging (fMRI, interevent-interval spike histograms) are also insensitive to the time structures often present in neural activity. The idea that disease states represent a loss of complexity rather than regularity has led to call for a radical revision of physiological analyses, and to a critique of the homeostatic principles that have guided explanations of physiological systems for most of the twentieth century (West, 2010). The claim is that homeostasis emphasizes Gaussian effects where negative feedback loops act to reduce variability and clamp systems at equilibrium and average rates, and has led to a “fix the number” approach to disease (Buchman, 2006), where attempts are made to restore the system to a fixed mean value. However, treatments based on this approach can lead to worsening rather than an improvement of conditions (Buchman, 2006; West, 2010). In contrast, complexity suggests variability and long-term correlations rather than quiescence at equilibrium, and that averages and central tendencies are replaced by the slope of power law functions. While this has offered important insight that has already had beneficial practical applications clinically (Mutch et al., 2000), the critiques of homeostasis that have been made are to some extent directed at a straw man that has developed as homeostasis has erroneously been reduced to its simplest form of the negative feedback control of fixed set-points (West, 2010). In introducing the concept of homeostasis Cannon (1932) said: “The word does not imply something set and immobile, a stagnation. It means a condition—a condition which may vary, but which is relatively constant.” Ashby (1958) also suggested that any controller or adaptive system needs a “requisite” level of variability to cope with the variable demands it may face, an idea embodied physically in his mechanical homeostat. Homeostasis is thus not synonymous with negative feedback, but can include internal feedback, feedforward prediction, parametric feedback, and hierarchical control that can reflect marked changes at lower levels, including positive feedback and amplification of perturbations, to maintain higher level functions. Many aspects considered “non-homeostatic” and new terms (e.g., homeodynamics, homeorhesis) were thus encompassed by the original view of homeostasis.

The loss of complexity rather than regularity as a marker in several disease conditions (West, 2010), and that physiological variability is correlated over different time scales and may reflect the degree of complexity of a system and the degree of its control and adaptability, are all important insights from analyses of criticality that have potentially significant implications for neurophysiological analyses. While variability is gradually being recognized (see Soltesz, 2006), its nature and relevance remain unclear at the neuronal or network level. We currently lack the insight into its underlying mechanisms to experimentally manipulate variability without also evoking changes in mean values (Aradi and Soltesz, 2002), assuming this is possible at all, making it difficult to relate experimental effects to changes in variance. Variability could reflect random noise, changes introduced by plasticity associated with different contexts or the history of the system (i.e., state-dependent differences), or actively programmed intrinsic variation in system or cell parameters that allow the system to select variable responses to variable inputs (Ashby, 1958, 1962). An important consideration is noise-induced variability: random noise can degrade or enhance signals (Smeal et al., 2010), whereas with the long range correlations associated with complexity order arises from noise. Whether noise is random or reflects long-range correlated signals is thus of significance to experimental analyses. In the former case it can be eliminated by averaging, and the variance decreases as the sample size increases. In the latter case the sample mean does not necessarily reduce to the population mean as the sample size increases (as expected by the central limit theorem), but can increase or decrease as more values are measured or the scale of the measurement is altered. A change in the mean does not necessarily reflect a change in the underlying processes, a basic tenet of experimental analyses of neuronal and other networks. Variance can also increase as additional data is analyzed. Thus, the mean and variance obtained over a certain scale will not be sufficient to characterize neuronal network parameters if networks are complex systems, and the analysis of signal fluctuations inspired by analyses of criticality may be more important than the characterization of their mean or variance. Consideration of this to neuronal network analyses would necessitate a marked change in experimental design (see Stumpf and Porter, 2012). This includes a focus on variability, which has often been filtered out in various ways by the design, selection, or interpretation of experimental data (see below), as well as demands on the data set.

## Complexity in movement and central pattern generating networks

Fractal or multifractal scaling has also been demonstrated in human locomotion, (Hausdorff et al., 1997). In healthy adults there are changes in gait (stride interval) that fluctuate about a mean value with long-range correlations that suggest that stride intervals are related to those occurring hundreds of strides earlier. This effect is independent of the speed of walking and is suggestive of a fractal property where stride interval is regulated over multiple gait cycles. These correlations were abolished when subjects walked to a metronome-controlled beat, suggesting a supraspinal influence on the fractal walking pattern, although the mechanisms underlying this effect are unknown. Scaling exponents differed between children and adults of different ages, and also in adults with varying severity of neurological disorders (Parkinson's disease, Huntington's disease, motor neuron (MN) disease), where walking became more random as long-range correlations were lost (see Hausdorff, 2009). This is highlighted as being indicative of different underlying mechanisms in the normal and abnormal motor systems, which on its own is a far from surprising conclusion.

What can the correlations of activity patterns over a wide range of frequencies say about the underlying effects? In a simple form movement needs pools of neurons controlling flexion and extension at different joints and limbs on the left and right sides of the body, and some way of regulating the activity in these pools to allow a co-ordinated global output. Flexion/extension and left/right coordination in the limbs occurs over various frequencies, both at the behavioral and at the cellular levels. The design principle revealed by the basic walking pattern, while not negating it as a useful or important parameter, does not tell you how or even why the system works the way it does, just what the system does: we again need insight into the mechanisms of these correlations. Correlations in step cycle duration have been mimicked in a modeling study of a spinal cord central pattern generator (CPG; see Hausdorff et al., 2001). A spinal CPG is a neuronal network that coordinates the patterned motor output to the muscles required for locomotion. In general terms a CPG consists of separate half-centers composed of specific cell classes and interactions that control antagonistic muscle groups (e.g., flexors and extensors). The half-centers are coupled by some form of mutually inhibitory connection that ensures their antiphasic activity. This was outlined over a century ago without consideration of the underlying cellular mechanisms (Brown, 1911). While there is some insight into the organization of these networks, the details are incomplete for even the simplest spinal CPG (lamprey; see for example Grillner et al., 2005; where despite the mantra that the network has been “defined experimentally,” much of the claimed experimental data does not exist and the characterization claimed is in fact a hypothesis; see Parker, 2006, 2010 for discussion).

Although details of actual spinal networks were not included in the modeling study of Hausdorff et al., the variability in the modeled motor output is of interest. Experimental analyses of actual spinal central pattern generating networks *in vitro* in response to pharmacological activation in lower vertebrate and mammalian systems (“fictive locomotion”) assume regular activity as the norm, and this has influenced analytical strategies that seek to confirm this assumption. For example, in a study that compared glutamate-evoked fictive activity in the isolated lamprey spinal cord with locomotor activity in swimming animals only regular activity was considered for analysis (it was stated that “the sequences selected for analysis in each preparation were those that appeared least variable”; Wallen and Williams, 1984). The selection was necessary because fictive activity is often irregular (Ayers et al., 1983; Parker et al., 1998; Parker and Bevan, 2007), and data are often searched for regions of regular activity or preparations are taken until one shows regular (“normal”) activity (it could be claimed that the quality of fictive activity shown is directly proportional to the number of experiments performed). This is a reflection of the belief that activity should be regular to resemble normal locomotion and that variability in the pattern is somehow abnormal, which counters the findings of the Hausdorff et al. (1997). If the situation is the same as the heartbeat, where regular interbeat intervals were also erroneously assumed to be the norm (Goldberger, 1996; Ivanov et al., 1999), it should instead be considered if rather than a model of normal function, highly regular fictive locomotor activity actually reflects a pathological loss of complexity. This could reflect the reliance for the activation of fictive locomotion on a fixed level of tonic bath applied glutamate receptor agonists, an approach that ignores the normal spatial and temporal variability of glutamate release. It could also reflect the removal of descending inputs from the brain and sensory feedback (see Cohen et al., 1996). This will reduce the interaction of the spinal CPG with other components of the motor system, an effect that can lead to a loss of complexity in dynamical systems (Pincus, 1994). That assumptions based on experimental analyses of fictive activity in isolated spinal cords needs re-consideration has been raised by Li et al. (2009) in the tadpole spinal cord, albeit not in terms of dynamical systems effects. Fictive locomotion may reflect a generic state (mathematically in the interior of a region corresponding to a given behavior): systems in such a state are considered structurally stable (Bienenstock and Lehmann, 1998) and do not show high susceptibility to external influences. Support for this is that when evoked by NMDA receptor activation, fictive locomotion in lamprey is less susceptible to modification by sensory feedback or descending inputs from the brain (see Fagerstedt and Ullén, 2001). Fictive locomotor activity is also more variable with the brain attached than in the isolated spinal cord, which is suggested to reflect the greater dynamical stability of the intact system (Cohen et al., 1996; Wang and Jung, 2002).

## Spinal cord networks as model systems for complexity analyses

The shift from regular to irregular activity with long-term correlations suggested by analyses of complexity in various systems highlights the need to consider the role of complexity in spinal central pattern generating networks where features relevant to criticality have been ignored or avoided (e.g., CPG variability during fictive locomotion). Spinal cord networks have a long history as model systems for understanding integrative function in the nervous system, and thus could provide useful models in which to attempt to relate system outputs and fractal analyses of network and cellular processes. The output (either fictive or actual) is simple to measure, and there is a wealth of cellular data that even though it has not yet offered an explanation of even simpler system outputs, does at least show the potential to address system outputs in cellular terms (Buchanan, 2001; Brownstone and Bui, 2010; Jankowska and Edgley, 2010; Roberts et al., 2010). These systems also emphasize the necessity of considering lower-level mechanisms in higher-level analyses rather than treating them as black-boxes full of hidden variables. Consider the simplified system in Figure 1, which reflects current views of the basic architecture of a spinal cord CPG network.

The two identical networks (half-centers) can control antagonistic muscle groups (e.g., flexor-extensor, left-right) through the sequential activation of MNs. The E neurons provide the excitatory drive to other neurons within each half-center, and receive inhibitory input from the I neurons. The crossing inhibitory and excitatory neurons (CI and CE, respectively) are the focus of this example as they have relatively direct assumed functional effects, alternation and synchronization of activity, respectively. Spinal cord networks have both types of neuron: the inhibitory class has dominated in concepts of spinal cord locomotor network schemes, but the relative roles of the two types of cell remain unclear (Kjaerulff and Kiehn, 1997; Berg et al., 2007). The basic idea is that given some tonic excitation, one side (e.g., the left side) becomes activated, the E neurons drive activity in MN on that side, and typically in conceptual schemes of this sort the CI neurons. This allows left side activity while ensuring the inhibition of right side activity. There will then be some burst termination factor that will end activity in the E cells (for this illustration these won't be detailed, but both cellular and synaptic mechanisms have been identified that could contribute): this will terminate activity of neurons in the active half-center and thus remove the CI drive. The previously silent right hand side can then become active through the tonic excitation and identified cellular rebound mechanisms that allow an escape from inhibition (Roberts and Tunstall, 1990). Given some tonic background excitation this system can generate repetitive cycles of antiphasic activity. However, the same output would be generated with crossed excitation from the CE to the inhibitory neurons within each half-center (I) that in turn feedforward to inhibit the E cells. Not only could both of these schemes generate the same output but both schemes would also exhibit the same effect to pharmacological blockade of inhibition, the routine approach used to show mutual inhibition between two sides and which is largely taken as support for crossed inhibition. Conversely, the inhibition between half-centers may not lead to alternating activity, but can lead to synchronous activity depending on the functional properties of the inhibitory connection (Elson et al., 2002). These examples illustrate the problem of inferring function from a consideration of higher-level analyses of outputs alone, as it cannot separate the markedly different organizations of crossed inhibition or excitation, and also the difficulties of inferring functional roles from a description of network motifs, of which reciprocal inhibition is arguably one of the prime examples. In terms of designing an artificial system either approach would work, but for intervening in the actual system the appropriate components would need to be considered (or their relative effects). If you tried to artificially mimic the effect of crossing neurons pharmacologically, say in a patient in which they had been lost or damaged, then you would need different approaches to substitute for the appropriate transmitter system (inhibitory or excitatory) and in a way that mimics the properties needed to evoke the functional effect desired. And if you wanted to modify the effects of the existing coupling between the two sides then in the crossed excitation case it may be better to follow an approach that targets excitation rather than inhibition.

Despite its advantages for experimental analyses, there have been few analyses of critical effects in spinal cord networks. Examples of this approach are Jung and Wang (2003) who showed long-term correlations for the burst interval during fictive locomotion in the lamprey, effects that were lost when inputs from the brainstem were removed, and Chang et al. (2004) who showed fractal correlations in bladder control that was lost in spinal cord injury; see also Rodríguez et al. (2011). While these analyses have only characterized the system output, they illustrate that useful insight for experimental analyses can be generated from statistical approaches. The loss of long-range correlations when fictive locomotor activity was examined after the spinal cord was isolated from the brain obviously suggests a role for either the feedforward descending pathway to the spinal cord alone, or the feedback interaction from the spinal cord to the brain, and highlights the need to avoid the tacit or expressed claims in several systems that fictive activity is a direct correlate of normal behavior (cf. Ayers et al., 1983; Li et al., 2009; see Parker, 2010). This matches the observation that fractal patterns in human gait are abolished when walking to a metronome beat, an input that will constrain the descending input to the spinal cord (Hausdorff et al., 1997), and suggests a conserved functional arrangement at the level of the spinal cord and its descending systems.

Importantly, these effects raise questions of components that are not “hidden” variables, but that are open to being addressed experimentally. Criticality has been examined at the behavioral, network, cellular, and synaptic levels in different systems (see Werner, 2010 and references therein) but not at all levels in a single system, thus making it difficult to make direct links between the different observations. Effects at the behavioral level (e.g., swimming, walking) can be compared with fictive locomotion in the isolated brainstem-spinal cord (to remove sensory inputs) and in the isolated spinal cord (to remove descending inputs; see Jung and Wang, 2003), allowing a separation of spinal locomotor network and sensory/descending influences on the long-range correlations in locomotor behavior, and analyses of what aspects of the spinal cord-sensory-brain feedforward or feedback loops are important for generating fractal effects on locomotion. These feedback loops could then suggest motifs underlying the network output and any critical phenomena. The analysis can then move to successively lower levels to examine the functional effects occurring in these motifs and also evidence for further examples of critical phenomena [e.g., fluctuations in the amplitude of timing of summed activity in locomotor network neurons, in the spiking in identified classes of cells (network neuron, sensory, and descending neurons; Buchanan, 2001), and of fluctuations in the amplitude and interval of synaptic inputs, which can be taken to the next level by comparing effects from identified monosynaptic connections (sensory, network, or descending; Parker, 2007)].

Some way will be needed of linking between effects as without this we will just be cataloguing and correlating effects at different levels. The correlative approach, where single component effects are examined at one level and manipulations of this effect are correlated with changes at the network or behavioral output, has not been sufficient to claim understanding of basic network function (Dudai, 2004; Parker, 2006, 2010). These correlations rely on or introduce several logical errors (Parker, 2006), and they will not be sufficient to identify the mechanisms underlying the development of critical effects. A focus on network motifs may be the best initial approach, as various levels can be peeled back to reveal the necessary components underlying criticality, and when these have been identified analyses can proceed to both higher and lower levels to identify the functional properties and translations that are needed for the generation or emergence of critical effects (if they are present). This will require changes in routinely used experimental designs in order to examine evidence for long-range correlations indicative of criticality (for example, the sufficiency of biological data sets to support power-law relationships (Stumpf and Porter, 2012; Varotsos et al. (2011), assumptions of linearity, stationarity, and equilibrium-like conditions and probabilistic averaging methods over certain scales (e.g., mean, variance, power spectra analyses) will not be suitable for non-stationary and non-equilibrium systems), while from the theoretical side acceptance that lower-level properties should be considered in dynamical systems approaches rather than dismissed as hidden, simple, enslaved, or abstracted components).

## Conclusions

While it remains an open question whether the nervous system is critical (e.g., Bedard et al., 2006; Werner, 2010), neuronal networks do contain features of critical systems, and as outlined above, it seems appropriate that they should function at the critical phase and be organized according to local rules that mean it is neither locked into a rigid functional state nor susceptible to random activity (Turing, 1950). The highlighting of variability in physiological systems; that correlations over different time scales may reflect the degree of complexity, control, and adaptability of a system; the move away from the caricature of homeostasis as equal to negative feedback driven clamping of a set point; the implications of fractal effects to the mean and variance in data sets; and the loss of complexity not regularity as a marker in several disease conditions are all important features derived from dynamical systems approaches that should inform experimental analyses of neuronal networks. These analyses may in turn help to identify the mechanisms that constitute a SOC system (Flyvbjerg, 1995), and thus to address whether SOC is a fundamental network property, an attempt to reduce neurophysiological processes to a level that can be formalized and described mathematically, an epiphenomena of statistical analyses applied to systems of multiple interacting components, or a reflection of the fractal nature of (and response to) natural signals (Voss and Clarke, 1975; Teich et al., 1990, 1997).

Many experimental and analytical approaches can be followed in network analyses. Given our level of understanding it is hard to say that any approach is not potentially useful, but there is a sense of competition between analyses. Relations between levels in a dynamical system entail changes of scale, display new properties, and obey new laws (Anderson, 1972; Werner, 2007): changes in scale thus define new ontologies. This brings in the philosophical problem of emergence (Kim, 1999), and has led to claims that knowledge of higher functions can be decoupled from studies of cellular components, that lower-level properties are enslaved and can be abstracted away to provide quantitative descriptions of system-level effects (Haken, 2002, 2006; Werner, 2007). If this were the case then one would expect higher-level processes to be relatively unaffected by changes in lower level properties. This is obviously not the case; drugs, anesthetics, alcohol, all act on lower level properties, and all alter or disrupt higher level processes precisely because they affect specific cellular and synaptic events. A focus on the macroscopic level finds macroscopic effects. To say that this makes that lower-level properties irrelevant begs the question. Dynamical systems approaches do, however, offer tools for describing complex systems, and these phenomenological descriptions can provide predictive diagnostic markers (e.g., heart disease or neurological disorders; see above). While the limits of even detailed cellular analyses are often highlighted by these approaches and cannot be ignored, quantitative descriptions of system-level effects fail to explain how system-level behavior is generated, a requirement for any rational intervention. If, as claimed, circular interactions between lower-level mechanisms and higher-level functions drive brain activity (Haken, 2002; Freeman, 2009), then insight is needed at both levels for genuine understanding. Thus, explaining how higher function “emerges out of electrical impulses and neurochemistry” (Chialvo et al., 2008) is impossible if electrical impulses and neurochemistry are excluded. The difficulty lies in knowing how to incorporate the data from different analyses: as outlined above it is not enough to correlate experimental manipulations of single effects to network or higher function (Dudai, 2004; Parker, 2006, 2010). There need not be competition between analyses as microlevel information will not necessarily replace or negate abstract descriptions. An obvious example is the Hodgkin–Huxley equation for the squid action potential (Hodgkin and Huxley, 1952), arguably the prime example from neuroscience of how phenomenological analyses can explain function despite the lack of direct analysis of the underlying mechanisms. Although simplifying assumptions were made (that the axon was a perfect cylinder), the equation predicted (and still does) effects in a range of systems and provided insight that led to lower-level details being filled in when technology allowed (Hodgkin and Huxley originally suggested “activation particles” as the mechanism of how voltage changes alter membrane conductance). This additional lower-level details on channel gating and structure (Hille, 2001; Jiang et al., 2003) did not alter the power of their original equations, but revealed details of the underlying mechanisms that added to our understanding of cellular excitability and its modification.

Rather than competition, a complementary approach where the strengths and weaknesses of lower and higher-level approaches are considered and attempts are made to close the gap between them and provide a more complete description. To stop at the level of statistical analyses leads to the assumption that description equals explanation (Wigner, 1964). It may be impossible to link effects across levels in emergent systems, but we need to know much more about properties and translations at each level before we follow functionalist-like suggestions that network outputs cannot be explained in terms of lower-level properties. If they either cannot (or need not) then we need to understand why this is, and the implications this has to our ability to intervene rationally in the system.

While discussions of the merits of different analytical approaches are becoming more sophisticated (e.g., Ivanov et al., 2009; Gao et al., 2006; Rodríguez et al., 2011) and the issue of the specific applications to biological systems has been raised (“extended criticality”; Bailly and Longo, 2008), it is important to work to develop a dialogue between the analytical and experimental sides so that the explanatory gap that makes it difficult to translate between the experimental and theoretical sides can be narrowed. Even if higher-level function can be divorced from lower-level mechanisms, it cannot harm the analyses to place effects in neurobiological contexts. Given the difficulties of explaining how groups of neurons interact in networks to generate specific outputs and the limitations of even the latest experimental approaches to help address this issue (Parker, 2006, 2010), there is a lot to recommend abstract approaches. Even though biological details are lost in these analyses through various assumptions (for example, binary effects, nearest neighbor or all-to-all interactions, and homogeneity), suggesting how they are related to neurobiological mechanisms opens up the possibility of examining effects at the lower-level. Yeh et al. (2010) offer a good example: in discussing the problems of employing maximal entropy models by highlighting the differential success of these models in the retina and cortex, they describe a third-order coupling constant as the situation where “two input neurons would drive a third neuron over threshold only when both inputs were simultaneously active.” The former term is opaque in terms of neurobiology, the latter statement clear and prescriptive. At a minimum, some clarity in what various dynamic patterns represent in terms of biological substrates would be useful. For example, chaotic effects can be claimed to represent few variables that act interdependently through global rather than local non-linear positive and negative feedback loops; critical effects reflect many variables acting interdependently through local rather than global feedback loops; random effects reflect many independent variables that lack feedback mechanisms; and periodic effects reflect a few variables with linear interactions. Just this level of detail can be enough to place effects in a neurobiological context and inspire experimental investigations.

The benefit can go both ways as neurobiological principles may help to improve model performance (see Yeh et al., 2010). Given the massive increase in computational demands placed on models when even modest increases in network size or parameter additions are made (see Nowotny et al., 2007; Yeh et al., 2010), insight that constrains the theoretical models would be beneficial. An example of this is given by Levina et al. (2007): models can replicate neuronal avalanche behavior in cortical slices that have been used to support criticality (Beggs and Plenz, 2003), but in the abstract models these only occurred when parameter values were fixed precisely. Levina et al. show that incorporating simple biologically inspired dynamical properties to the modeled connections they were able to generate self-organized critical behavior that resembled experimental effects and thus overcame the limitations of previous models. Without knowledge of these simple principles of synaptic dynamics this would not have been possible, and there may be similar biological details that advance other higher level analyses.

It may seem remarkable that nervous systems and artificial systems show the same network organization when examined from a dynamical systems approach (see Bullmore and Sporns, 2009), and that the brain, which is given special status, can be described in relation to something so mundane. However, ignoring lower-level properties begs the question that no special property is present at lower levels. It should be realized that even single neurons do not lack detail, but are themselves complex dynamic structure with markedly heterogeneous properties that differ under different contexts of network activity (patterns, frequency, duration) and in terms of the history of the system. Despite claims to the contrary, the neuron cannot be considered as simple and repetitive (cf. Zhigulin, 2004; West, 2010). There may not be a ghost in the machine, but the devil may be in the detail.

### Conflict of interest statement

The authors declare that the research was conducted in the absence of any commercial or financial relationships that could be construed as a potential conflict of interest.
